# Early pneumonia and timing of antibiotic therapy in patients after nontraumatic out-of-hospital cardiac arrest

**DOI:** 10.1186/s13054-016-1191-y

**Published:** 2016-02-01

**Authors:** Kristian Hellenkamp, Sabrina Onimischewski, Jochen Kruppa, Martin Faßhauer, Alexander Becker, Helmut Eiffert, Mark Hünlich, Gerd Hasenfuß, Rolf Wachter

**Affiliations:** 1 Clinic for Cardiology & Pneumology/Heart Center, University Medical Center Göttingen, Robert-Koch-Straße 40, 37075 Göttingen, Germany; 2 Department of Medical Statistics, Georg-August-University Göttingen, Humboldtallee 32, Göttingen, 37073 Germany; 3 Institute for Diagnostic and Interventional Radiology, Georg-August-University Göttingen, Robert-Koch-Straße 40, Göttingen, 37075 Germany; 4 Institute for Medical Microbiology, Georg-August-University Göttingen, Kreuzbergring 57, Göttingen, 37075 Germany

**Keywords:** Out-of-hospital cardiac arrest, Pneumonia, Postcardiac arrest syndrome, Therapeutic hypothermia, Infection, Antibiotic therapy

## Abstract

**Background:**

While early pneumonia is common in patients after out-of-hospital cardiac arrest (OHCA), little is known about the impact of pneumonia and the optimal timing of antibiotic therapy after OHCA.

**Methods:**

We conducted a 5-year retrospective cohort study, including patients who suffered from OHCA and were treated with therapeutic hypothermia. ICU treatment was strictly standardized with defined treatment goals and procedures. Medical records, chest radiographic images and microbiological findings were reviewed.

**Results:**

Within the study period, 442 patients were admitted to our medical ICU after successfully resuscitated cardiac arrest. Of those, 174 patients fulfilled all inclusion and no exclusion criteria and were included into final analysis. Pneumonia within the first week could be *confirmed* in 39 patients (22.4 %) and was *confirmed or probable* in 100 patients (57.5 %), without a difference between survivors and non-survivors (37.8 % vs. 23.1 % *confirmed pneumonia*, p = 0.125). In patients with *confirmed pneumonia* a tracheotomy was performed more frequently (28.2 vs. 12.6 %, p = 0.026) compared to patients without *confirmed pneumonia*. Importantly, patients with *confirmed pneumonia* had a longer ICU- (14.0 [8.5-20.0] vs. 8.0 [5.0-14.0] days, p < 0.001) and hospital stay (23.0 [11.5-29.0] vs. 15.0 [6.5-25.0] days, p = 0.016).

A positive end expiratory pressure (PEEP) > =10.5 mbar on day 1 of the hospital stay was identified as early predictor of *confirmed pneumonia* (odds ratio 2.898, p = 0.006). No other reliable predictor could be identified.

Median time to antibiotic therapy was 8.7 [5.4-22.8] hours, without a difference between patients with or without *confirmed pneumonia* (p = 0.381) and without a difference between survivors and non-survivors (p = 0.264). Patients receiving antibiotics within 12 hours after admission had a shorter ICU- (8.0 [4.0-14.0] vs. 10.5 [6.0-16.0] vs. 13.5 [8.0-20.0] days, p = 0.004) and hospital-stay (14.0 [6.0-25.0] vs. 16.5 [11.0-27.0] vs. 21.0 [17.0-28.0] days, p = 0.007) compared to patients receiving antibiotics after 12 to 36 or more than 36 hours, respectively.

**Conclusions:**

Early pneumonia may extend length of ICU- and hospital-stay after OHCA and its occurrence is difficult to predict. A delayed initiation of antibiotic therapy in OHCA patients may increase the duration of the ICU- and hospital-stay.

**Electronic supplementary material:**

The online version of this article (doi:10.1186/s13054-016-1191-y) contains supplementary material, which is available to authorized users.

## Background

The annual incidence of out-of-hospital cardiac arrest (OHCA) is around 35 per 100,000, of which only about 10 % survive until hospital discharge [1–3]. The high mortality rate of patients who initially achieve return of spontaneous circulation (ROSC) can be attributed to a pathophysiological process which is called postcardiac arrest syndrome [4]. As part of the postcardiac arrest syndrome, ischemia/reperfusion activates immunological pathways contributing to an increased risk of infection [5–7]. Indeed, infectious complications occur frequently after OHCA, especially in the early phase [8, 9]. However, because of confounding factors, appropriate and prompt diagnosis of infection after OHCA remains challenging.

For instance, therapeutic hypothermia, which is recommended in survivors of OHCA to improve their neurological outcome [2, 4], complicates the diagnosis of infection because changes in body temperature cannot be considered. Moreover, whether or not hypothermia itself may impair the immune system and increase infection rates is still a matter of debate [9–11]. Remarkably, while infections are common and difficult to diagnose after OHCA, little is known about the impact of timing of antibiotic therapy in those patients.

We hypothesized that, after OHCA, early pneumonia is common, has prognostic impact, and can be predicted by clinical variables. Thus, we conducted a retrospective cohort study aiming to analyze the incidence and the impact of early pneumonia, to identify possible early predictors of pneumonia, and to analyze the impact of timing of antibiotic therapy on the occurrence of pneumonia and outcome after OHCA.

## Methods

### Study design and study population

We performed a 5-year retrospective cohort study including patients admitted to our medical ICU in a university hospital between 1 January 2010 and 31 December 2014 after successful resuscitation for OHCA. The study was approved by the local ethics committee of the university hospital of Göttingen, Germany.

### Definitions and standard care on our ICU

All original chest radiographies performed within the first 7 days of hospitalization were analyzed by a single experienced radiologist (MF) for new or progressive infiltrates. This radiologist was blinded to all clinical data (e.g., microbiological findings) and only interpreted the original chest X-ray images in batches. Chest radiographies were classified into one of four categories—new or progressive infiltrates were: (category 1) definitely present, (category 2) probably present, (category 3) unlikely, or (category 4) excluded. According to published criteria of the International Sepsis Forum Consensus Conference [12] we distinguished between confirmed pneumonia and probable pneumonia. Confirmed pneumonia was diagnosed when new radiographic infiltrates on chest radiography were present (category 1) in combination with clinical suspicion and the isolation of a pulmonary pathogen from a lower respiratory tract sample. Bacteriological findings considered to cause pneumonia are summarized in Additional file 1. For microbiological confirmation, semiquantitative Gram staining was performed on each specimen. Semiquantitative cultures were processed according to standard culture procedures. The microbiological investigation was defined positive if in the Gram staining two or more (oil immersion (×1000) per field) bacteria were found and the culture was positive. We excluded *Staphylococcus epidermidis*, *Enterococcus* species, and *Streptococcus agalactiae* to be a causative agent for pneumonia. Clinical suspicion was assumed when at least one of the following criteria was present: purulent sputum; auscultation findings suspicious for pneumonia; or hypoxemia (Partial pressure of oxygen in arterial blood (PaO_2_) /Fraction of inspired oxygen (FiO_2_) <240). Probable pneumonia was diagnosed without a microbiological confirmation in the presence of a new or progressive infiltrate on chest radiography (category 1) when pneumonia was clinically present (defined as purulent sputum plus at least one of the criteria of suspicious auscultation findings or hypoxemia). We also diagnosed a probable pneumonia when pneumonia was clinically present (see earlier), a suspicious pulmonary pathogen from a lower respiratory tract sample could be isolated (see earlier), and chest radiography revealed a probable infiltrate (category 2). Early pneumonia was defined as confirmed pneumonia or probable pneumonia within 7 days.

Treatment of OHCA patients admitted to our ICU is strictly standardized. Treatment goals and procedures and further definitions are provided in Additional file 2.

### Data analysis and statistical methods

Data were collected and analyzed using Microsoft Excel, SPSS version 22.0 (SPSS, Inc., Chicago, IL, USA) and R version 3.1.2 (The R Foundation for Statistical Computing, Vienna, Austria). Proportions are expressed as percentages and as absolute numbers. Continuous variables are given as the median value (25th–75th percentiles). Statistical tests used in this study were two-tailed and used a significance level of 0.05. Categorical variables were compared using the Fisher’s exact test. Comparisons of continuous variables were performed with the Mann–Whitney U test. To test for correlations between continuous variables, the Spearman correlation coefficient (*r*) was calculated. To identify possible predictors of pneumonia we dichotomized numerous variables (including patient demographic characteristics, cardiovascular risk factors, parameters of resuscitation, laboratory values indicating infection, hemodynamic and respiratory parameters) according to the occurrence of pneumonia. Since we wanted to identify early markers of pneumonia, only parameters available within the first 3 days after admission were analyzed. Variables included in our definition of pneumonia diagnosis (e.g., purulent sputum) were not considered. Since all patients were treated with therapeutic hypothermia, fever was also not considered. However, since the median time until rewarming was 46.8 (43.0–50.1) hours, we included the occurrence of post-hypothermia fever (for definition see Additional file 2) in the analysis. Receiver operating characteristic (ROC) analysis was performed for baseline parameters significantly different between patients with and without pneumonia, and the area under the curve (AUC) was calculated with regard to the occurrence of confirmed pneumonia. Youden Index quantification was used to identify the optimal cutoff value for the prediction of pneumonia. For the optimal cutoff value, the specificity, sensitivity, and negative and positive predictive values were calculated and univariate logistic regression analysis was performed to obtain the odds ratio (OR) with the corresponding 95 % confidence intervals (95 % CIs). In-hospital death was analyzed as the primary outcome parameter. Secondary outcome parameters were time to extubation (days), length of ICU stay (days), length of hospital stay (days), and need for tracheotomy. Time to extubation was analyzed for all patients extubated within their ICU stay, when no reintubation or tracheotomy was necessary.

## Results

### Patient population

**Fig. 1:**
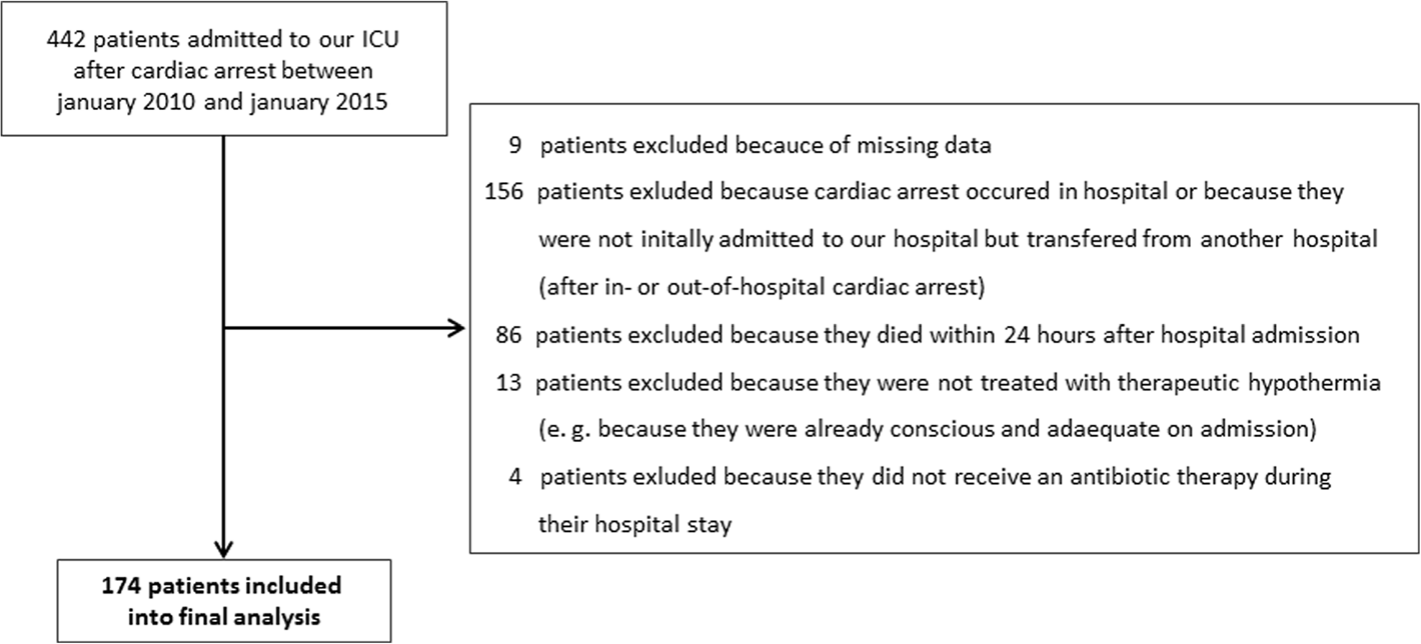
Flow chart of patient enrollment and exclusion

Between January 2010 and January 2015, 442 patients were admitted to our medical ICU after primarily surviving cardiac arrest. Of those, 174 patients were included into the final study analysis. Figure 1 shows a flow chart of patient enrollment and exclusion. Patient characteristics, diagnostics, and therapeutic procedures are summarized in Table 1 (dichotomized according to survivors and nonsurvivors).

**Table 1:** Baseline characteristics, cardiovascular risk factors, parameters of resuscitation, diagnostic work-up, diagnosis, and therapy for all patients and dichotomized according to survivors and nonsurvivors

Variable	All patients (*n* = 174)	Survivors (*n* = 114)	Nonsurvivors (*n* = 60)	*p* value
Age (years)	69.0 (57.0–77.0)	64.5 (53.0–73.0)	74.0 (64.5–80.0)	**<0.001**
Sex category = male	135 (77.6 %)	92 (80.7 %)	43 (71.7 %)	0.185
Cardiovascular risk factors				
Hypertension	96 (55.2 %)	63 (55.3 %)	33 (55.0 %)	1.000
Diabetes mellitus	39 (22.4 %)	23 (20.2 %)	16 (26.7 %)	0.344
Adipositas	28 (16.1 %)	18 (15.8 %)	10 (16.7 %)	1.000
Known CVD	40 (23.0 %)	20 (17.5 %)	20 (33.3 %)	**0.023**
Cardiopulmonary resuscitation				
Free interval (minutes), *n* = 146	5.0 (0.0–10.0)	2.0 (0.0–10.0)	7.5 (2.0–10.0)	**0.006**
First rhythm				
VT	3 (1.7 %)	3 (2.6 %)	0 (0.0 %)	0.552
VF	113 (64.9 %)	89 (78.1 %)	24 (40.0 %)	**<0.001**
Asystole	34 (19.5 %)	9 (7.9 %)	25 (41.7 %)	**<0.001**
PEA	17 (9.8 %)	8 (7.0 %)	9 (15.0 %)	0.110
Other or unknown	7 (4.0 %)	5 (4.4 %)	2 (3.3 %)	1.000
Time to ROSC (minutes), *n* = 146	24.5 (15.0–35.0)	20.0 (14.0–31.0)	30.0 (17.8–35.0)	**0.023**
Witnessed aspiration	22 (12.6 %)	15 (13.2 %)	7 (11.7 %)	1.000
Diagnostic workup				
Coronary angiography	149 (85.6 %)	106 (93.0 %)	43 (71.7 %)	**<0.001**
Diagnosis/suspected cause of cardiac arrest				
STEMI	57 (32.8 %)	47 (41.2 %)	10 (16.7 %)	**0.001**
NSTEMI	34 (19.5 %)	23 (20.2 %)	11 (18.3 %)	0.843
Pulmonary embolism	5 (2.9 %)	3 (2.6 %)	2 (3.3 %)	1.000
Primary arrhythmia	26 (14.9 %)	14 (12.3 %)	12 (20.0 %)	0.186
Other or unknown	52 (29.9 %)	27 (23.7 %)	25 (41.7 %)	**0.016**
Therapy				
Impella or IABP	38 (21.8 %)	29 (25.4 %)	9 (15.0 %)	0.126
PCI	80 (46.0 %)	61 (53.5 %)	19 (31.7 %)	**0.007**

For definitions, see Methods. Data presented as absolute numbers (percentages) or medians (25th–75th percentile). *n* refers to the number of patients with available data. *p* values were calculated by the Mann–Whitney U test or Fisher’s exact test

*CVD* coronary vascular disease, *IABP* intraaortic balloon pump, *NSTEMI* non-ST-elevation myocardial infarction, *PEA* pulseless electrical activity, *PCI* percutaneous coronary intervention, *ROSC* return of spontaneous circulation, *STEMI* ST-elevation myocardial infarction, *VF* ventricular fibrillation, *VT* ventricular tachycardia

For variables that are statistically different betwen survivors and non-survivors given *p*-values are highlighted by bold characters

In nonsurvivors, the median time to death was 5.5 (3.5–9.5) days.

### Incidence and impact of early pneumonia

Overall, 591 chest X-ray scans were performed within the first 7 days (mean 0.49 X-ray images per patient per day) and evaluated with regard to the presence of infiltrates. In 92 images (15.6 %) infiltrates could be excluded, in 136 images (23.0 %) infiltrates were rated unlikely, and in 205 images (34.7 %) infiltrates were rated probably present. In 158 X-ray images (26.7 %) infiltrates were definitely confirmed.

Early pneumonia within 7 days could be confirmed in 39 patients (22.4 %). One hundred patients (57.5 %) had either a confirmed or a probable early pneumonia within 7 days. Of note, 32 patients (18.4 %) had a confirmed pneumonia and 93 patients (53.4 %) had either a confirmed or probable pneumonia within 5 days.

**Fig. 2:**
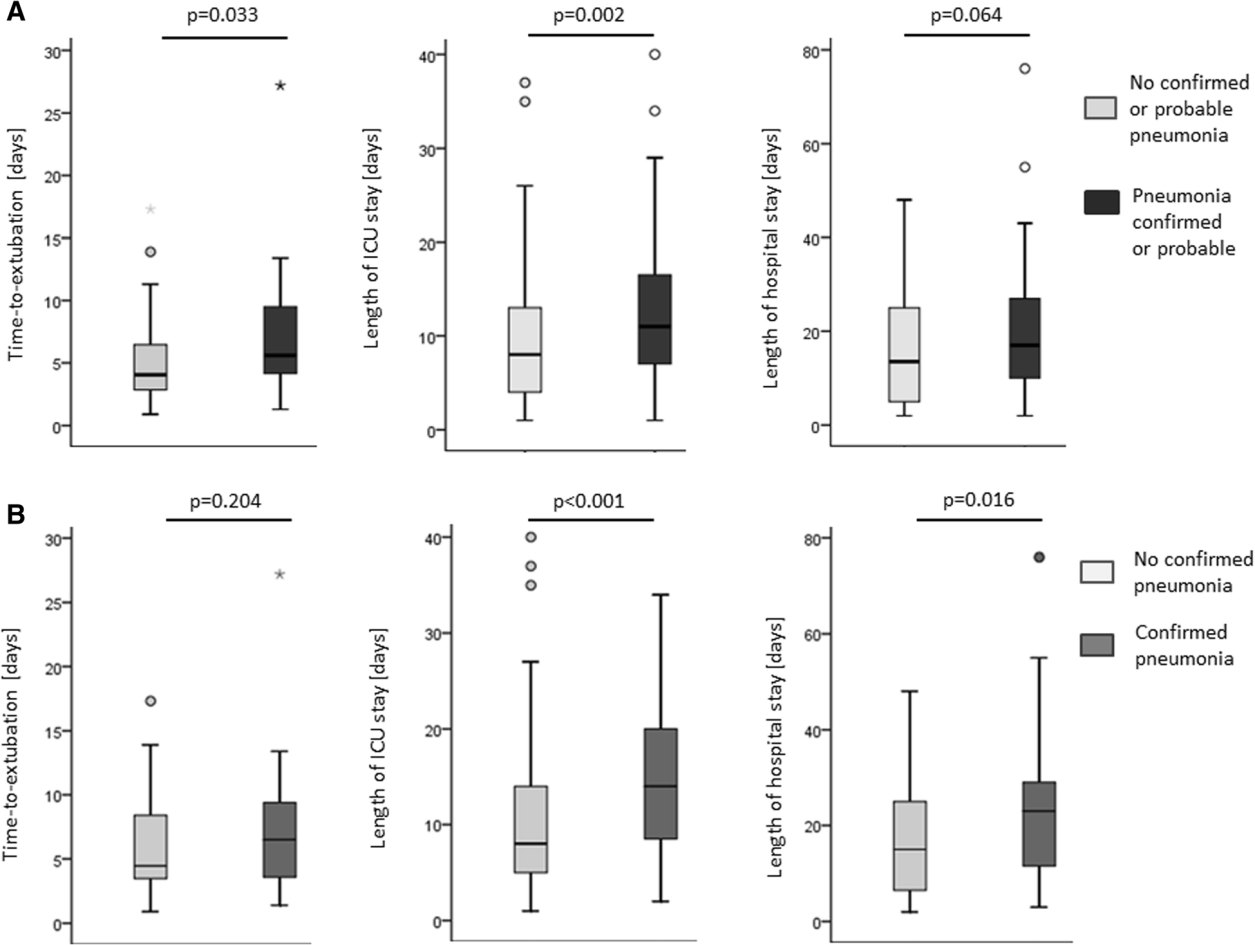
Impact of early pneumonia on time to extubation, length of ICU stay, and length of hospital stay. **a** Comparison between patients with or without confirmed or probable pneumonia. **b** Comparison between patients with or without confirmed pneumonia

In all patients diagnosed to have a confirmed pneumonia within 7 days, we evaluated which of the three required criteria (clinical suspicion, presence of infiltrates on chest X-ray scan, and bacteriological finding considered to cause pneumonia; see Methods) was the last to become positive. The last criterion becoming positive was a bacteriological finding in 31 of 39 patients (79.5 %), an infiltrate on chest X-ray scan in 4 of 39 patients (10.3 %), and clinical suspicion in 0 of 39 patients (0 %). In four patients (10.3 %) an infiltrate on chest X-ray scan and a bacteriological finding considered to cause pneumonia were found on the same day. Of note, in none of the four patients in which a positive chest X-ray image was the last criterion becoming positive was a chest X-ray scan performed the day before. This may indicate a potential delay in diagnosis for those patients, caused by the fact that chest radiography was not performed daily in our ICU. There was no difference regarding the incidence of confirmed pneumonia between survivors and nonsurvivors (37.8 % vs. 23.1 %, *p* = 0.125). Similarly, confirmed or probable pneumonia was found as often in survivors as in nonsurvivors (60.5 % vs. 51.7 %, *p* = 0.333). Patients with confirmed pneumonia had a longer ICU stay (14.0 (8.5–20.0) vs. 8.0 (5.0–14.0) days, *p* <0.001) and hospital stay (23.0 (11.5–29.0) vs. 15.0 (6.5–25.0) days, *p* = 0.016) compared with patients without pneumonia, while there was no difference regarding the time to extubation (6.5 (3.6–9.4) vs. 4.5 (3.5–8.4) days, *p* = 0.204) (Fig. 2). The rate of tracheotomy was higher in patients with confirmed pneumonia compared with patients without pneumonia (28.2 vs. 12.6 %, *p* = 0.026).

Likewise, in patients with confirmed or probable pneumonia, the time to extubation (5.6 (4.2–9.5) vs. 4.1 (2.9–6.5) days, *p* = 0.033) and the length of ICU stay (11.0 (7.0–16.5) vs. 8.0 (4.0–13.0) days, *p* = 0.002) were increased. The length of hospital stay (17.0 (10.0–27.0) vs. 13.5 (5.0–25.0), *p* = 0.064) and the rate of tracheotomy (18.0 vs. 13.5 %, *p* = 0.533) did not differ between patients with or without confirmed or probable pneumonia.

### Predictors of pneumonia

Numerous variables available within 3 days after hospital admission (Table 2 and Additional file 3) were dichotomized according to pneumonia diagnosis. Patients with confirmed pneumonia and patients with confirmed or probable pneumonia had higher levels of the maximum positive end-expiratory pressure (PEEP) on day 1 compared with patients without pneumonia. Maximal PEEP correlated negatively with the minimal PO_2_/FiO_2_ value of the same day (day 1, *r* = −0.614; day 2, *r* = −0.629; day 3, *r* = −0.650; *p* <0.001 for all). ROC analysis for the PEEP level on day 1 with regard to the occurrence of confirmed pneumonia and sensitivity and specificity analysis are presented in Additional file 4. Overall, 73.0 % of patients had a maximum PEEP value on day 1 below 10.5 mbar which was identified as optimal cutoff. Maximum PEEP values ≥10.5 mbar on day 1 were associated with an increased risk for the occurrence of confirmed pneumonia in univariate logistic regression analysis (OR 2.621 (95 % CI 1.370–5.014), *p* = 0.004).

**Table 2:** Comparison of patients with and without confirmed pneumonia – all parameters available within 3 days

	All patients	No confirmed pneumonia	Confirmed pneumonia	*p* value
	(*n* = 174)	(*n* = 135)	(*n* = 39)	
Age (years)	69.0 (57.0–77.0)	68.0 (57.0–76.5)	72.0 (60.0–77.5)	0.303
Sex category = male	135 (77.6 %)	104 of 135 (77.0 %)	31 of 39 (79.5 %)	0.830
Cardiovascular risk factors				
Smoking	65 (37.4 %)	51 of 135 (37.8 %)	14 of 39 (35.9 %)	0.493
Hypertension	96 (55.2 %)	72 of 135 (53.3 %)	24 of 39 (61.5 %)	0.465
Diabetes mellitus	39 (22.4 %)	33 of 135 (24.4 %)	6 of 39 (15.4 %)	0.281
Adipositas	28 (16.1 %)	23 of 135 (17.0 %)	5 of 39 (12.8 %)	0.627
Known coronary vascular disease	40 (23.0 %)	29 of 135 (21.5 %)	11 of 39 (28.2 %)	0.393
Cardiopulmonary resuscitation				
Free interval (minutes)	5.0 (0.0–10.0)	5.0 (0–10.0)	3.0 (0–10.0)	0.928
First rhythm				
Ventricular tachycardia	3 (1.7 %)	3 of 135 (2.2 %)	0 of 39 (0.0 %)	1.000
Ventricular fibrillation	113 (64.9 %)	88 of 135 (65.2 %)	25 of 39 (64.1 %)	1.000
Asystole	34 (19.5 %)	27 of 135 (20.0 %)	7 of 39 (17.9 %)	1.000
Pulseless electrical activity	17 (9.8 %)	12 of 135 (8.9 %)	5 of 39 (12.8 %)	0.540
Other or unknown	7 (4.0 %)	5 of 135 (3.7 %)	2 of 39 (5.1 %)	0.654
Time to ROSC (minutes)	24.5 (15.0–35.0)	25.0 (15.0–35.0)	22.0 (15.5–34.8)	0.666
Witnessed aspiration	22 (12.6 %)	18 of 135 (13.3 %)	4 of 39 (10.3 %)	0.787
Laboratory values				
CRP (mg/l) on admission (day 1), *n* = 170	3.0 (2.0–10.4)	3.0 (2.0–8.9)	2.9 (2.0–20.9)	0.915
CRP (mg/l) on day 2, *n* = 173	24.0 (8.6–50.7)	23.1 (8.7–48.1)	25.3 (7.0–52.7)	0.943
CRP (mg/l) on day 3, *n* = 169	124.1 (89.6–166.0)	124.2 (93.5–159.2)	123.6 (81.6–197.5)	0.896
WBC (10^3^/μl) on admission (day 1), *n* = 174	15.5 (10.7–19.1)	15.2 (10.6–18.2)	16.6 (11.2–21.0)	0.237
WBC (10^3^/μl) day 2, *n* = 174	12.7 (9.2–17.5)	12.9 (9.6–17.4)	10.3 (9.1–17.1)	0.295
WBC (10^3^/μl) day 3, *n* = 170	12.3 (9.1–15.6)	12.5 (9.3–15.9)	11.2 (8.4–14.9)	0.204
PCT (μg/l) day 1, *n* = 121^a^	0.1 (0.1–0.6)	0.1 (0.1–0.5)	0.2 (0.1–1.1)	0.312
PCT (μg/l) day 2, *n* = 83^a^	1.9 (0.5–7.7)	2.0 (0.5–10.2)	1.2 (0.5–2.7)	0.271
PCT (μg/l) day 3, *n* = 89^a^	1.7 (0.5–6–1)	1.6 (0.6–6.1)	3.0 (0.5–6.0)	0.642
Lactate (mmol/l) on admission, *n* = 172	3.7 (2.2–6.8)	5.4 (3.3–7.9)	4.7 (3.2–6.6)	0.301
Respiration				
PO_2_/FiO_2_ minimum on day 1	152 (100–228)	152 (100–230)	161 (109–211)	0.891
PO_2_/FiO_2_ minimum on day 2	165 (121–222)	160 (117–220)	167 (132–220)	0.502
PO_2_/FiO_2_ minimum on day 3	160 (126–205)	160 (127–206)	155 (128–188)	0.479
PEEP maximum (mbar) on day 1	8.5 (7.0–11.0)	8.0 (7.0–10.0)	10.0 (8.0–12.0)	**0.014**
PEEP maximum (mbar) on day 2	9.0 (7.0–11.0)	8.0 (7.0–10.0)	10.0 (7.5–12.0)	0.114
PEEP maximum (mbar) on day 3	9.0 (7.0–12.0)	8.0 (7.0–12.0)	9.0 (7.0–11.5)	0.603
Hemodynamics				
Vasopressor dosage^b^ (μg/minute) on day 1	16.0 (8.0–32.0)	16.0 (8.0–30.0)	14.0 (9.5–33.5)	0.712
Vasopressor dosage^b^ (μg/minute) on day 2	19.0 (10.0–32.0)	20.0 (10.0–32.0)	18.0 (10.0–33.0)	0.983
Vasopressor dosage^b^ (μg/minute) on day 3	16.0 (9.0–32.0)	16.0 (9.0–32.0)	16.0 (9.5–38.0)	0.941
Volume infusion (l) on day 1	4.7 (3.1–7.0)	4.5 (3.0–6.9)	5.1 (3.2–7.6)	0.469
Volume infusion (l) on day 2	7.0 (5.0–9.8)	7.3 (5.3–9.8)	6.8 (4.3–9.3)	0.396
Volume infusion (l) on day 3	4.5 (3.4–6.4)	4.4 (3.4–6.4)	4.8 (3.9–6.1)	0.347
Infection and antibiotics				
Time to antibiotics (hours)	8.7 (5.4–22.8)	8.5 (5.4–22.4)	9.0 (6.0–26.9)	0.381
Post-hypothermia fever, *n* = 135^c^	75 of 135 (55.6 %)	54 of 101 (53.5 %)	21 of 34 (61.8 %)	0.431

Data presented as absolute numbers (percentages) or median (25th–75th percentile). *n* refers to the number of patients with available data

We also tested whether or not there was a difference of any of the presented parameters between patients with or without confirmed or probable pneumonia. Data are presented in Additional file 3

^a^Missing data, because PCT values are not measured routinely every day in our ICU

^b^Intravenous vasopressors were given in order to maintain a mean arterial pressure (MAD) of >65 mmHg. Norepinephrine was used as vasopressor of first choice; epinephrine was used when a second vasopressor was necessary to maintain MAD. When a second vasopressor was necessary, numbers in the table reflect the dosage of both vasopressors. The number gives the highest dosage used on each day

^c^Missing data are caused by patients already being dead at this time point

*CRP* C-reactive protein, *PCT* procalcitonin *PEEP* positive end expiratory pressure, *ROSC* return of spontaneous circulation, *WBC* white blood cell count, *PaO*_*2*_ */FiO*_*2*_ Partial pressure of oxygen in arterial blood [mmHg] / Fraction of inspired oxygen

For variables that are statistically different betwen patients with and without confirmed pneumonia given *p*-values are highlighted by bold characters.

### Timing of antibiotic therapy

All patients included in this study received antibiotic therapy (Fig. 1). In 166 patients (95.4 %), piperacillin tazobactam was given as initial antibiotic therapy, either alone (68.1 %) or in combination with clarithromycin (31.9 %). Five patients (2.9 %) received amoxicillin clavulanic acid or ampicillin sulbactam combined with clarithromycin as initial antibiotic therapy. Meropenem was given to two patients (1.2 %), and one patient (0.6 %) was treated with ceftriaxone in combination with erythromycin.

**Fig. 3:**
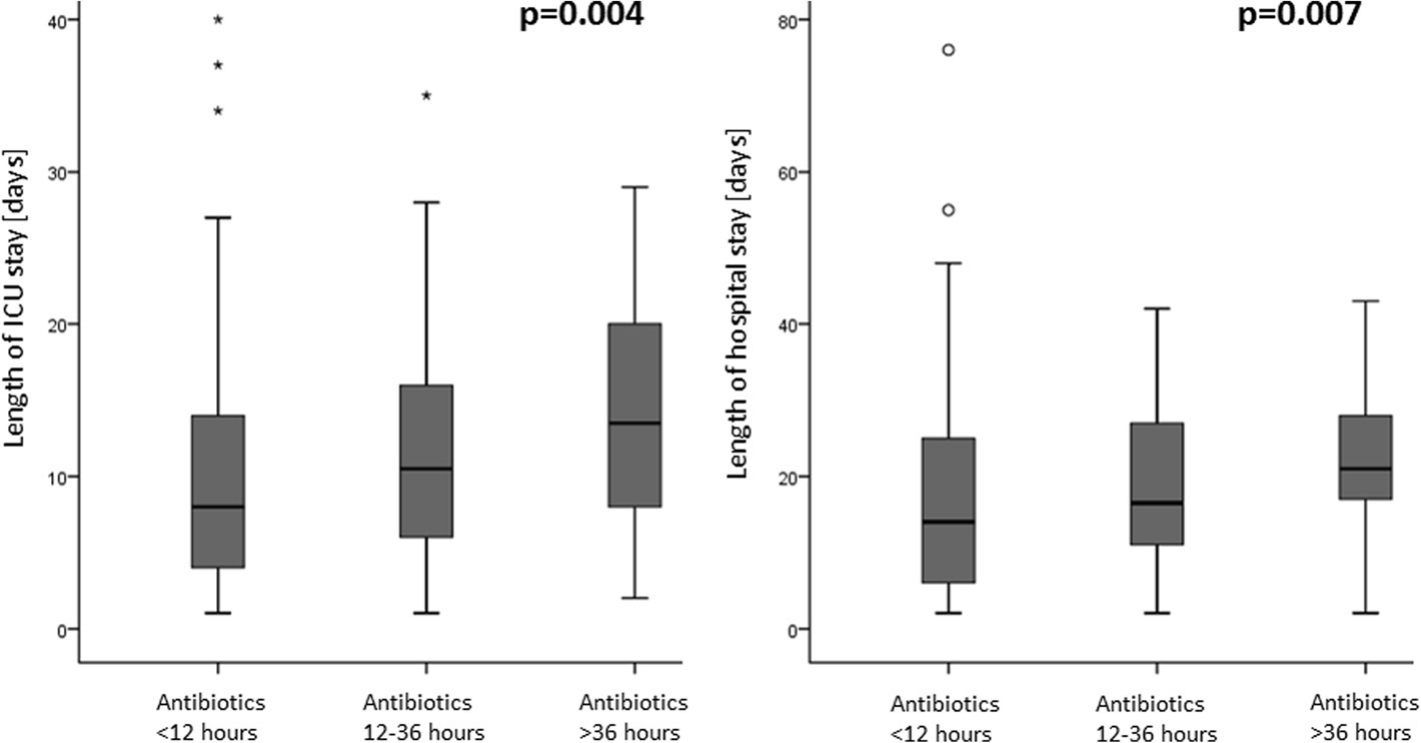
Influence of timing of antibiotic therapy on the length of the ICU stay and the length of hospital stay

The median time until an antibiotic therapy was initiated was 8.7 hours (5.4–22.8 hours) without a difference between patients with or without confirmed pneumonia (8.9 (5.4–29.0) vs. 8.6 (5.5–21.9) hours, *p* = 0.497) and patients with or without confirmed or probable pneumonia (7.7 (5.1–24.2) vs. 11.3 (6.6–22.5) hours, *p* = 0.263). Likewise, there was no difference between survivors and nonsurvivors (10.4 (5.4–26.1) vs. 8.0 (5.8–16.0) hours, *p* = 0.264). However, patients needing a tracheotomy in the clinical course (28 patients, 16.1 %) had a later initiation of antibiotic therapy (22.5 (6.5–36.9) vs. 8.1 (5.4–20.0) hours, *p* = 0.029). Furthermore, we found a weak but significant correlation between the time until an antibiotic therapy was started and the duration of the ICU stay (correlation coefficient *r* = 0.17, *p* = 0.026) and the hospital stay (correlation coefficient *r* = 0.17, *p* = 0.026). For further analysis, we grouped all patients into one of three classes according to timing of antibiotic therapy: antibiotics were given within 12 hours (category I, 101 patients, 58.0 %), 12–36 hours (category II, 51 patients, 29.3 %) or after more than 36 hours (category III, 22 patients, 12.6 %), respectively. When grouped into categories, length of ICU stay (8.0 (4.0–14.0) vs. 10.5 (6.0–16.0) vs. 13.5 (8.0–20.0) days for category I, II, and III, respectively; *p* = 0.004) and length of hospital stay (14.0 (6.0–25.0) vs. 16.5 (11.0–27.0) vs. 21.0 (17.0–28.0) days for category I, II, and III, respectively; *p* = 0.007) was shorter in patients receiving early antibiotics (Fig. 3).

## Discussion

Our study has three main findings: 1) early pneumonia occurs frequently in patients treated with therapeutic hypothermia after OHCA, but is difficult to predict; 2) early pneumonia after OHCA may increase the rate of tracheotomy and extend the length of the ICU and hospital stay; and 3) a delayed initiation of antibiotic therapy after OHCA may increase the length of the ICU and hospital stay.

### Incidence of early pneumonia

We found an incidence of 22.4 % for confirmed pneumonia and 57.5 % for confirmed or probable pneumonia within 7 days after hospital admission. In the literature, early pneumonia is reported to occur in 27.5–65 % of patients treated with therapeutic hypothermia after OHCA [8, 9, 13–18]. The large heterogeneity across different studies might be caused by differences regarding the definition of early pneumonia. Moreover, diagnosis of pneumonia is challenging, since common criteria such as body temperature and leukocytosis may be affected by hypothermia and can therefore not be employed. Another challenging fact regarding the definition of pneumonia is that interobserver agreement in the interpretation of chest radiographs for pneumonia is reported to be moderate [19], indicating that confirmation of infiltrates may not always be definite. This limitation may be even worse in the setting of bedside radiography on the ICU. Therefore, in our study, only patients with a chest X-ray scan categorized “infiltrates definitely present” (as rated by an experienced radiologist) could have confirmed pneumonia. This may explain why only relatively few patients had confirmed pneumonia in our study, if compared with previous reports where no information considering the reliability of infiltrate detection on chest radiographies were given [8, 9, 13–18].

### Impact of early pneumonia

In agreement with most previous reports [8, 9, 13], in our study confirmed pneumonia and confirmed or probable pneumonia were diagnosed as often in survivors as in nonsurvivors. In contrast, MacLaren et al. [17] reported higher incidences of pneumonia in OHCA patients with a good neurological recovery or Nielsen et al. [14] reported higher incidences in patients alive at the time of follow-up. However, these counterintuitive findings likely relate to the fact that nonsurvivors may not survive long enough to develop (and to have diagnosed) pneumonia. Indeed, in our study, the median time to death in nonsurvivors was only 5.5 (3.5–9.5) days. Another possible explanation for the absence of an impact of early pneumonia on survival in our study may be the probable adequacy of antibiotic therapy (nearly all patients received piperacillin–tazobactam).

Nonetheless, our data suggest prognostic relevance of pneumonia, as indicated by a longer ICU and hospital stay compared with patients without pneumonia. Moreover, patients with confirmed pneumonia had a higher rate of tracheotomy. Consistently, previous studies reported an increased length of mechanical ventilation [9, 13], an increased length of ICU stay [9, 13], and increased rate of tracheotomy in patients with early pneumonia [13].

### Predictors of pneumonia

Since diagnosis of pneumonia is difficult in the setting of therapeutic hypothermia after OHCA and our data and previous reports [9, 13] suggest a prognostic relevance of early pneumonia, we tried to identify predictors of pneumonia which could be assessed within the first 3 days after hospital admission. Out of 39 variables (Table 2) compared between patients with and without confirmed pneumonia, only the PEEP level on day 1 was different between the two groups. The same was the case for patients with confirmed or probable pneumonia if compared with patients without pneumonia (Additional file 3). Higher PEEP levels are usually applied if oxygen diffusion is impaired and impaired oxygen diffusion is part of the pathophysiology of pneumonia, which makes our observation biologically plausible. Hence, higher PEEP may well be indicative of early pneumonia, but may also be a contributor to the pathogenesis because it leads to bacterial translocation in an animal model [20]. With respect to the retrospective design of our study, we therefore cannot say whether higher PEEP levels are a cause or a consequence of early pneumonia. This question remains unanswered and requires further investigations (e.g., a prospective randomized trial). However, in daily clinical practice, an elevated PEEP on day 1 may be of limited usefulness regarding the prediction of pneumonia, since the observed difference between PEEP values was only moderate and with overlapping CIs.

### Timing of antibiotic therapy

Since we failed to identify reliable early predictors of pneumonia, we analyzed whether or not timing of antibiotic therapy is of prognostic relevance after OHCA. Importantly, we observed no difference regarding the duration until an antibiotic therapy was initiated between survivors and nonsurvivors. In contrast, Davies et al. [21] reported improved survival when antibiotics were given within the first 7 days following OHCA. They therefore conclude that early antibiotics improve survival after OHCA. However, in their study the median delay between ICU admission and first dose of antibiotics was 2.17 days. Since, the mortality was highest in the early phase, some patients may not have survived long enough to receive antibiotics (rather than dying because they did not receive antibiotics). Indeed, when the analysis was restricted to patients surviving (at least) until day 3, no survival difference between patients with or without antibiotic therapy was found [21]. In our study, all patients received antibiotics and antibiotic therapy was initiated after a median time of 8.7 hours. Since, in this early phase, infection may in most cases not be present, such an early start of antibiotics must be considered as prophylactic treatment. Recently, a registry study reported a reduced incidence of pneumonia in patients receiving antibiotic prophylaxis after OHCA [15]. In contrast, we and others [9] found no association between the timing of antibiotic treatment and the incidence of pneumonia. In the report by Gagnon et al. [15], antibiotic prophylaxis was given in 33.5 % of patients. Interestingly, this percentage varied widely (0–100 %) between different centers, demonstrating the urgent need for more evidence for or against prophylactic antibiotics after OHCA.

In our study, patients needing a tracheotomy during their ICU stay had a later initiation of antibiotic therapy. This finding may indicate a more disturbed respiratory status and a more severe clinical course in those patients. Accordingly, patients receiving antibiotics within 12 hours had a shorter ICU stay and hospital stay compared with patients with a delayed antibiotic therapy.

### Limitations

While our data may favor an early start of antibiotic therapy in OHCA patients treated with hypothermia, some potential limitations need to be mentioned. First, our study was a single-center study performed on a medical ICU. Thereby, we cannot assure that results are valid for different sites (e.g., for surgical or neurological ICUs). Second, our study focused on early pneumonia and we did not collect data on pneumonia occurring after 7 days, which is hospital acquired and may be predicted by different factors (e.g., age and diabetes) [22]. Moreover, in the literature, the definition of early pneumonia is not homogeneous: other reports used a 5-day cutoff value [8] to define early pneumonia, which would have resulted in a less than 20 % lower number of patients with confirmed pneumonia in our study. Third, the present study had a retrospective design reporting empirical evidence in favor of an early start of antibiotic therapy. However, because of the retrospective design, causality between observed differences cannot be proven and confounding factors may be present. On the other side, as a strength of our study, antibiotic therapy was very homogeneous (>95 % piperacillin tazobactam) compared with previous reports where antibiotic therapy was either more heterogeneous [21] or not reported [15]. Therefore, in our study, it is likely that differences regarding the length of ICU and hospital stay are caused by the timing of antibiotic therapy rather than by the selected antibiotic. Furthermore, in contrast to a previous registry-based report [15], in our study ICU treatment was strictly standardized and detailed information regarding the reliability of infiltrate detection on chest radiographies, the definition of pneumonia, laboratory values, as well as respiratory and hemodynamic parameters can be provided.

## Conclusion

Early pneumonia occurs frequently in patients treated with therapeutic hypothermia after OHCA and may extend length of ICU stay, and length of hospital stay. Importantly, diagnosis of pneumonia in the setting of hypothermia after OHCA is challenging since common criteria cannot be taken into consideration (e.g., fever), are difficult to interpret (e.g., infiltrates on chest radiographies), or are available only with delay (microbiological findings). Only a PEEP value on day 1 above 10.5 mbar was found to be an early predictor of confirmed pneumonia; other (more reliable) predictors could not be identified. Since our data show that a delayed initiation of antibiotic therapy after OHCA may increase the length of ICU stay and hospital stay, prospective trials are warranted to confirm these findings and to identify the optimal point in time to initiate antibiotic therapy.

## Key messages

- Early pneumonia occurs frequently in patients treated with therapeutic hypothermia after OHCA, but is difficult to predict.
- Early pneumonia after OHCA may increase the rate of tracheotomy and extend the length of the ICU and hospital stay.
- A delayed initiation of antibiotic therapy after OHCA may increase the length of the ICU and hospital stay.
- Prospective trials are warranted to identify the optimal point in time to initiate antibiotic therapy.

## Additional files

Additional file 1: **Table S1 presenting bacteriological microorganisms obtained from culture of endotracheal aspirates or bronchoalveolar lavages from patients with confirmed pneumonia.** (DOCX 15 kb)

Additional file 2: **Word document presenting additional materials and methods.** (DOCX 20 kb)

Additional file 3: **Table S2 presenting comparison of patients with and** ***without confirmed or probable pneumonia*** **– all parameters available within 3 days.** (DOCX 19 kb)

Additional file 4: **Figure S1 showing ROC analysis of the PEEP level on day 1 with regard to the prediction of confirmed pneumonia.** (DOCX 63 kb)

## Abbreviations

AUC: Area under the curve
CI: Confidence interval
OHCA: Out-of-hospital cardiac arrest
OR: Odds ratio
PEEP: Positive end-expiratory pressure
ROC: Receiver operating characteristic
ROSC: Return of spontaneous circulation
