# Supplementary material for: Early pneumonia and timing of antibiotic therapy in patients after nontraumatic out-of-hospital cardiac arrest
Source: Crit Care. 2016 Feb 1;20:31. doi: 10.1186/s13054-016-1191-y (PMC4736704; doi:10.1186/s13054-016-1191-y)
Supplement: Supplementary file 1 — Table S1 presenting bacteriological microorganisms obtained from culture of endotracheal aspirates or bronchoalveolar lavages from patients with confirmed pneumonia. (DOCX 15 kb) [file 13054_2016_1191_MOESM1_ESM.docx]

**Additional file 1**

**Table S1. Bacteriological micro-organisms obtained from culture of endotracheal aspirates or bronchoalveolar lavages from patients with *confirmed pneumonia*.**

| **Gram-positive bacteria** |  |
| --- | --- |
| Staphylococcus |  |
| Staphylococcus aureus | 17 (23.6%) |
| Staphylococcus (other or without further classification) | 2 (2.8%) |
| Streptococcus |  |
| Streptococcus pneumoniae | 6 (8.3%) |
| Streptococcus species (other or without further classification) | 3 (4.2%) |
| Gram-positive coccus without further classification | 2 (2.8%) |
| **Gram-negative bacteria** |  |
| Escherichia coli | 12 (16.7%) |
| Serratia species | 6 (8.3%) |
| Klebsiella pneumoniae | 4 (5.6%) |
| Klebsiella oxytoca | 3 (4.2%) |
| Raoultella ornithinolytica | 1 (1.4%) |
| Haemophilus influenzae | 4 (5.6%) |
| Enterobacter cloacae | 3 (4.2%) |
| Enterobacter aerogenes | 2 (2.8%) |
| Proteus mirabilis | 3 (4.2%) |
| Proteus vulgaris | 3 (4.2%) |
| Citrobacter koseri | 2 (2.8%) |
| Other | 1 (1.4%) |
