# Supplementary material for: Early pneumonia and timing of antibiotic therapy in patients after nontraumatic out-of-hospital cardiac arrest
Source: Crit Care. 2016 Feb 1;20:31. doi: 10.1186/s13054-016-1191-y (PMC4736704; doi:10.1186/s13054-016-1191-y)
Supplement: Supplementary file 2 — Word document presenting additional materials and methods. (DOCX 20 kb) [file 13054_2016_1191_MOESM2_ESM.docx]

**Additional file 2**

**Additional material and methods section**

**Definitions**

The *free interval* was defined as the duration from loss of consciousness until resuscitation was started, regardless whether resuscitation was started by bystanders or by healthcare professionals. *Time to return of spontaneous circulation (ROSC)* was defined as the duration from loss of consciousness until ROSC. *Post-hypothermia fever* was diagnosed when the patient had a body temperature >38°C within 48 hours after rewarming (which was also assumed when the intravascular cooling catheter (CoolGard System) was maintained for at least 48 hours after rewarming of the patient to a body temperature of 36.5°C due to active cooling of the device in order to avoid fever). *Time to antibiotic therapy* was defined as the time frame from admission to the hospital to initiation of antibiotic therapy.

**Standard care on our medical ICU**

Since this was a retrospective analysis, treatment decisions were made by the physicians caring for the patient according to current guidelines [1, 2] and not influenced by the study protocol. However, treatment of patients admitted to our ICU after primary survived out-of-hospital cardiac arrest is strictly standardized with defined therapy goals: intravenous vasopressors are given in order to maintain a mean arterial pressure (MAD) of >65 mm Hg [3]. Norepinephrine is used as vasopressor of first choice; epinephrine is used when a second vasopressor was necessary to maintain MAD. In cases of low cardiac output – as indicated by echocardiography or by hemodynamic measurements (e. g. using pulse contour analysis calibrated by trans-pulmonary thermodilution, PiCCO^TM^) – dobutamine is used as positive inotropic drug of first choice. Ventilator settings are adjusted in order to obtain a pO_2_ >80 mmHg, an oxygen saturation (SaO_2_) >90% and normocarbia [1, 2]. Principles of lung protective ventilation (tidal volume not exceeding 6 mL/kg (predicted) body weight and a plateau pressure not exceeding 30 cm H_2_O [4]) are applied whenever sufficient to maintain treatment goals [2]. All patients included into analysis were treated with therapeutic hypothermia. If not already started in the out-of-hospital setting, hypothermia was initiated immediately after ICU admission by rapid infusion of cold saline to achieve a target temperature of 33° to 34°C. To maintain target temperature for 24 hours, in most patients (160 of 174; 92%) an intravascular cooling catheter (CoolGard System) was used. In all other patients, target temperature was maintained with cold saline or external cooling. After 24 hours patients were rewarmed at a rate of 0.25°C/h to a core temperature of 36.5°C which was maintained for at least 12 hours. In cases of evidence of fever (indicated by cooling by the intravascular cooling catheter system), the CoolGard System was maintained with a target temperature of 36.5°C to a maximum period of 72 hours after rewarming.

**References (additional file 2)**

# Neumar RW, Nolan JP, Adrie C, Aibiki M, Berg RA, Böttiger BW, et al. [Post-cardiac arrest syndrome: epidemiology, pathophysiology, treatment, and prognostication. A consensus statement from the International Liaison Committee on Resuscitation (American Heart Association, Australian and New Zealand Council on Resuscitation, European Resuscitation Council, Heart and Stroke Foundation of Canada, InterAmerican Heart Foundation, Resuscitation Council of Asia, and the Resuscitation Council of Southern Africa); the American Heart Association Emergency Cardiovascular Care Committee; the Council on Cardiovascular Surgery and Anesthesia; the Council on Cardiopulmonary, Perioperative, and Critical Care; the Council on Clinical Cardiology; and the Stroke Council.](http://www.ncbi.nlm.nih.gov/pubmed/18948368) Circulation. 2008 Dec 2;118(23):2452-83.

# Deakin CD, Nolan JP, Soar J, Sunde K, Koster RW, Smith GB, et al. [European Resuscitation Council Guidelines for Resuscitation 2010 Section 4. Adult advanced life support.](http://www.ncbi.nlm.nih.gov/pubmed/20956049) Resuscitation. 2010 Oct;81(10):1305-52.

# [Sunde K](http://www.ncbi.nlm.nih.gov/pubmed/?term=Sunde%20K%5BAuthor%5D&cauthor=true&cauthor_uid=17258378), [Pytte M](http://www.ncbi.nlm.nih.gov/pubmed/?term=Pytte%20M%5BAuthor%5D&cauthor=true&cauthor_uid=17258378), [Jacobsen D](http://www.ncbi.nlm.nih.gov/pubmed/?term=Jacobsen%20D%5BAuthor%5D&cauthor=true&cauthor_uid=17258378), [Mangschau A](http://www.ncbi.nlm.nih.gov/pubmed/?term=Mangschau%20A%5BAuthor%5D&cauthor=true&cauthor_uid=17258378), [Jensen LP](http://www.ncbi.nlm.nih.gov/pubmed/?term=Jensen%20LP%5BAuthor%5D&cauthor=true&cauthor_uid=17258378), [Smedsrud C](http://www.ncbi.nlm.nih.gov/pubmed/?term=Smedsrud%20C%5BAuthor%5D&cauthor=true&cauthor_uid=17258378), et al. Implementation of a standardised treatment protocol for post resuscitation care after out-of-hospital cardiac arrest. [Resuscitation.](http://www.ncbi.nlm.nih.gov/pubmed/?term=Sunde+K%2C+Pytte+M%2C+Jacobsen+D%2C+et+al.+Implementation+of+a+standardised+treat-ment+protocol+for+post+resuscitation+care+after+out-of-hospital+cardiac+arrest.+Resuscitation+2007%3B73%3A29%E2%80%9339.) 2007 Apr;73(1):29-39.

# [Dellinger RP](http://www.ncbi.nlm.nih.gov/pubmed/?term=Dellinger%20RP%5BAuthor%5D&cauthor=true&cauthor_uid=18158437), [Levy MM](http://www.ncbi.nlm.nih.gov/pubmed/?term=Levy%20MM%5BAuthor%5D&cauthor=true&cauthor_uid=18158437), [Carlet JM](http://www.ncbi.nlm.nih.gov/pubmed/?term=Carlet%20JM%5BAuthor%5D&cauthor=true&cauthor_uid=18158437), [Bion J](http://www.ncbi.nlm.nih.gov/pubmed/?term=Bion%20J%5BAuthor%5D&cauthor=true&cauthor_uid=18158437), [Parker MM](http://www.ncbi.nlm.nih.gov/pubmed/?term=Parker%20MM%5BAuthor%5D&cauthor=true&cauthor_uid=18158437), [Jaeschke R](http://www.ncbi.nlm.nih.gov/pubmed/?term=Jaeschke%20R%5BAuthor%5D&cauthor=true&cauthor_uid=18158437), et al. Surviving Sepsis Campaign: international guidelines for management of severe sepsis and septic shock: 2008. [Crit Care Med.](http://www.ncbi.nlm.nih.gov/pubmed/?term=Dellinger+RP%2C+Levy+MM%2C+Carlet+JM%2C+European+Society+of+Intensive+Care+Medicine) 2008 Jan;36(1):296-327.
