# Supplementary material for: Early pneumonia and timing of antibiotic therapy in patients after nontraumatic out-of-hospital cardiac arrest
Source: Crit Care. 2016 Feb 1;20:31. doi: 10.1186/s13054-016-1191-y (PMC4736704; doi:10.1186/s13054-016-1191-y)
Supplement: Supplementary file 3 — Table S2 presenting comparison of patients with and without confirmed or probable pneumonia – all parameters available within 3 days. (DOCX 19 kb) [file 13054_2016_1191_MOESM3_ESM.docx]

Table S2. Comparison of patients with and without *confirmed or probable pneumonia* – all parameters available within 3 days.

|  | **All patients**  **(n=174)** | **No pneumonia**  **(n=74)** | **Confirmed or probable pneumonia^#^ (n=100)** | **P-value** |
| --- | --- | --- | --- | --- |
| Age [years] | 69.0 [57.0-77.0] | 70.0 [57.0-75.0] | 67.0 [57.5-77.0] | 0.995 |
| Sex category = male | 135 (77.6%) | 57 of 74 [77.0%] | 78 of 100 [78.0%] | 1.000 |
| **Cardiovascular risk factors** | | | | |
| Smoking | 65 (37.4%) | 26 of 74 (35.1%) | 39 of 100 (39.0%) | 0.637 |
| Hypertension | 96 (55.2%) | 44 of 74 (59.5%) | 52 of 100 (52.0%) | 0.358 |
| Diabetes mellitus | 39 (22.4%) | 19 of 74 (25.7%) | 20 of 100 (20.0%) | 0.462 |
| Adipositas | 28 (16.1%) | 12 of 74 (16.2%) | 16 of 100 (16.0%) | 1.000 |
| Known coronary vascular disease | 40 (23.0%) | 14 of 74 (18.9%) | 26 of 100 (26.0%) | 0.362 |
| **Cardiopulmonary resuscitation** | | | | |
| Free interval [min] | 5.0 [0.0-10.0] | 5.0 [0-10.0] | 3.5 [0-10.0] | 0.901 |
| First Rhythm  Ventricular tachycardia  Ventricular fibrillation  Asystole  Pulseless electrical activity  Other or unkown | 3 (1.7%)  113 (64.9%)  34 (19.5%)  17 (9.8%)  7 (4.0%) | 1 of 74 (1.4%)  47 of 74 (63.5%)  16 of 74 (21.6%)  8 of 74 (10.8%)  2 of 74 (2.7%) | 2 of 100 (2.0%)  66 of 100 (66.0%)  18 of 100 (18.0%)  9 of 100 (9.0%)  5 of 100 (5.0%) | 1.000  0.750  0.567  0.798  0.700 |
| Time-to-ROSC [min] | 24.5 [15.0-35.0] | 25.0 [15.0-35.0] | 21.3 [15.0-35.0] | 0.705 |
| Witnessed aspiration | 22 (12.6%) | 9 of 74 (12.2%) | 13 of 100 (13.0%) | 1.000 |
| **Laboratory values** | | | | |
| CRP [mg/l] on admission (day 1), n=170 | 3.0 [2.0-10.4] | 3.1 [2.0-10.1] | 2.9 [2.0-10.7] | 0.890 |
| CRP [mg/l] on day 2, n=173 | 24.0 [8.6-50.7] | 23.9 [8.6-51.2] | 24.0 [8.5-48.7] | 0.925 |
| CRP [mg/l] on day 3, n=169 | 124.1 [89.6-166.0] | 128.8 [88.2-186.8] | 123.0 [88.9-156.7] | 0.561 |
| WBC on admission (day 1), n=174 | 15.5 [10.7-19.1] | 14.0 [10.7-17.7] | 16.6 [10.7-20.2] | 0.087 |
| WBC day 2, n=174 | 12.7 [9.2-17.5] | 13.7 [9.5-18.5] | 12.1 [9.1-16.3] | 0.259 |
| WBC day 3, n=170 | 12.3 [9.1-15.6] | 12.5 [9.2-16.3] | 12.2 [9.2-15.5] | 0.564 |
| PCT [µg/l] day 1, n=121* | 0.1 [0.1-0.6] | 0.1 [0.1-0.5] | 0.1 [0.1-0.6] | 0.922 |
| PCT [µg/l] day 2, n=83* | 1.9 [0.5-7.7] | 3.4 [0.5-15.7] | 1.6 [0.6-4.4] | 0.224 |
| PCT [µg/l] day 3, n=89* | 1.7 [0.5-6-1] | 1.8 [0.6-9.2] | 1.7 [0.4-5.0] | 0.216 |
| Lactate on admission, n=172 | 3.7 [2.2-6.8] | 3.7 [2.5-7.4] | 3.5 [1.9-6.0] | 0.090 |
| **Respiration** | | | | |
| PO_2_/FiO_2_ min on day 1 | 152 [100-228] | 155 [103-254] | 152 [96-217] | 0.199 |
| PO_2_/FiO_2_ min on day 2 | 165 [121-222] | 168 [117-225] | 165 [122-217] | 0.919 |
| PO_2_/FiO_2_ min on day 3 | 160 [126-205] | 162 [118-211] | 158 [133-190] | 0.607 |
| PEEP max [mbar] on day 1 | 8.5 [7.0-11.0] | 8.0 [7.0-10.0] | 10.0 [8.0-12.0] | **0.006** |
| PEEP max [mbar] on day 2 | 9.0 [7.0-11.0] | 8.0 [7.0-10.0] | 9.5 [7.0-12.0] | 0.095 |
| PEEP max [mbar] on day 3 | 9.0 [7.0-12.0] | 8.0 [7.0-11.0] | 9.0 [7.0-12.0] | 0.205 |
| **Hemodynamics** | | | | |
| vasopressor dosage** [µg/min] on day 1 | 16.0 [8.0-32.0] | 12.0 [6.0-30.0] | 17.0 [9.0-32.0] | 0.342 |
| vasopressor dosage ** [µg/min] on day 2 | 19.0 [10.0-32.0] | 15.5 [9.0-32.0] | 20.0 [11.0-32.0] | 0.214 |
| vasopressor dosage ** [µg/min] on day 3 | 16.0 [9.0-32.0] | 13.0 [8.0-30.0] | 20.0 [10.0-33.0] | 0.182 |
| Volume infusion [l] on day 1 | 4.7 [3.1-7.0] | 4.7 [3.0-7.1] | 4.7 [3.2-6.9] | 0.879 |
| Volume infusion [l] on day 2 | 7.0 [5.0-9.8] | 6.8 [5.3-9.0] | 7.5 [5.0-10.0] | 0.454 |
| Volume infusion [l] on day 3 | 4.5 [3.4-6.4] | 4.4 [3.3-6.4] | 4.6 [3.6-6.2] | 0.534 |
| **Infection and antibiotics** | | | | |
| Time-to-antibiotics [hours] | 8.7 [5.4-22.8] | 11.3 [6.6-22.5] | 7.7 [5.1-24.2] | 0.263 |
| Post-hypothermia fever (n=135)° | 75 of 135 (55.6%) | 26 of 52 (50.0%) | 49 of 83 (59.0%) | 0.374 |

Abbreviations: Min refers to minutes; CRP, C-reactive protein; WBC, white blood cell count; PEEP, positive end expiratory pressure.

Units (if not given in the table): WBC: 10^3^/µl; Lactate: mmol/l.

Statistical methods: Data are presented as absolute numbers and percentages in parenthesis or medians with the corresponding 25th–75th percentile in square brackets; (n) refers to the number of patients with available data.

*Missing data, because Procalcitonin (PCT) values are not measured routinely every day on our ICU.

**intravenous vasopressors were given in order to maintain a mean arterial pressure (MAD) of >65 mm Hg. Norepinephrine was used as vasopressor of first choice; epinephrine was used when a second vasopressor was necessary to maintain MAD. When a second vasopressor was necessary numbers in the table reflect the dosage of both vasopressors. The number gives the highest dosage used on each day.

°Missing data are caused by patients already death at this time point.
