# Supplementary material for: Early pneumonia and timing of antibiotic therapy in patients after nontraumatic out-of-hospital cardiac arrest
Source: Crit Care. 2016 Feb 1;20:31. doi: 10.1186/s13054-016-1191-y (PMC4736704; doi:10.1186/s13054-016-1191-y)
Supplement: Supplementary file 4 — Figure S1 showing ROC analysis of the PEEP level on day 1 with regard to the prediction of confirmed pneumonia. (DOCX 63 kb) [file 13054_2016_1191_MOESM4_ESM.docx]

**Additional file 4**

Figure S1. ROC analysis of PEEP level on day 1 with regard to the prediction of *confirmed pneumonia.*


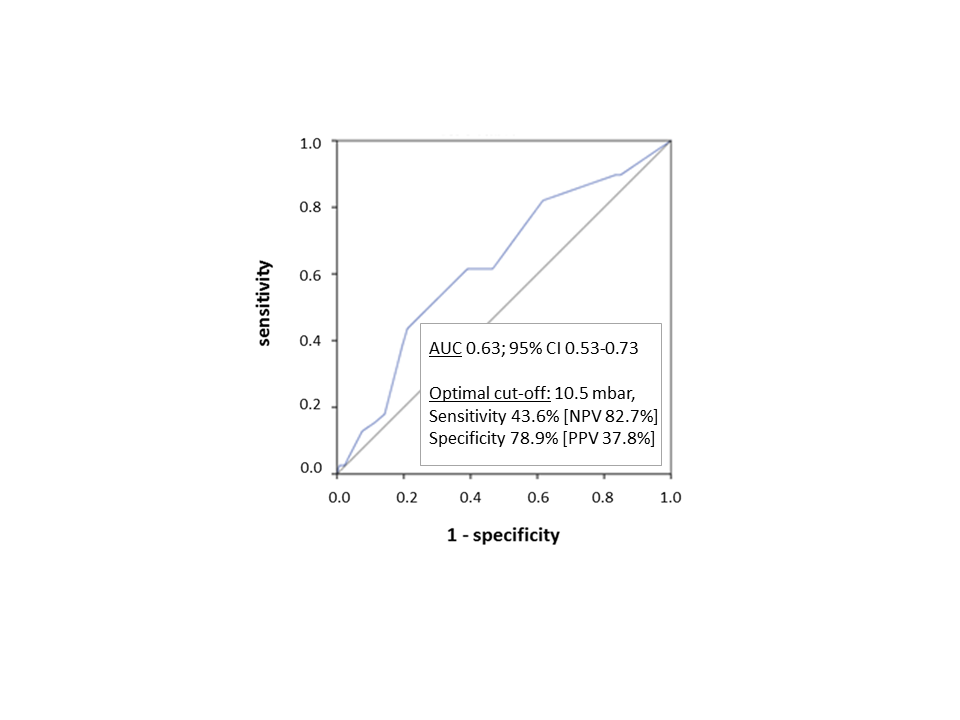


Abbreviations: ROC, receiver operating characteristics; PEEP, positive end expiratory pressure; AUC, area under the curve; CI, confidence interval; NPV, negative predictive value; PPV, positive predictive value.
